# Larger group sizes facilitate the emergence and spread of innovations in a group-living bird

**DOI:** 10.1016/j.anbehav.2019.10.004

**Published:** 2019-12

**Authors:** Benjamin J. Ashton, Alex Thornton, Amanda R. Ridley

**Affiliations:** a Centre for Evolutionary Biology, University of Western Australia, Perth, Australia; b School of Biological Sciences, University of Bristol, Bristol, UK; c Centre for Ecology and Conservation, University of Exeter, Penryn Campus, UK

**Keywords:** animal innovation, group size, pool of competence hypothesis

## Abstract

The benefits of group living have traditionally been attributed to risk dilution or the efficient exploitation of resources; individuals in social groups may therefore benefit from access to valuable information. If sociality facilitates access to information, then individuals in larger groups may be predicted to solve novel problems faster than individuals in smaller groups. Additionally, larger group sizes may facilitate the subsequent spread of innovations within animal groups, as has been proposed for human societies. We presented a novel foraging task (where a food reward could be accessed by pushing a self-shutting sliding door) to 16 groups of wild, cooperatively breeding Australian magpies, *Cracticus tibicen dorsalis*, ranging in size from two to 11 individuals. We found a nonlinear decline in the time taken for the innovative behaviour to emerge with increasing group size, and social information use facilitated the transmission of novel behaviour, with it spreading more quickly in larger than smaller groups. This study provides important evidence for a nonlinear relationship between group size and the emergence of innovation (and its subsequent transmission) in a wild population of animals. Further work investigating the scope and strength of group size–innovation relationships, and the mechanisms underpinning them, will help us understand the potential advantages of living in larger social groups.

## Highlights

• We investigated the relationship between group size and innovative behaviour.
• Innovative behaviour emerges disproportionately quickly with increasing group size.
• Social information use facilitates the transmission of novel behaviour.
• These results highlight potential advantages of living in large social groups.

The benefits of group living have been studied extensively for decades (Krause & Ruxton, 2002), and are often attributed to processes that help animals exploit resources more efficiently (e.g. social foraging; Galef and Giraldeau, 2001, Giraldeau and Caraco, 2000), or reduce risks from threats such as predators (Silk, 2007). More recently, a growing body of evidence is lending support to the hypothesis that larger group sizes may also facilitate the emergence of innovative solutions to novel problems (Krause et al., 2010, Liker and Bókony, 2009, Morand-Ferron and Quinn, 2011).

Much of the evidence supporting a positive relationship between group size and problem-solving performance comes from studies on humans (Clément et al., 2013, Laughlin et al., 2006). Evidence of this relationship in nonhuman animals is limited to a handful of studies, and has produced equivocal results (reviewed by Griffin & Guez, 2015). Some studies report positive effects of group size (Liker and Bókony, 2009, Morand-Ferron and Quinn, 2011), whereas others report negative effects (Griffin et al., 2013, Overington et al., 2009) or no effect (Thornton and Malapert, 2009a, Thornton and Samson, 2012).

A number of potential factors may generate a positive relationship between group size and the emergence of behavioural innovations. For instance, the presence of more group members may reduce neophobia and the need to invest in antipredator vigilance, facilitating the exploitation of novel foraging resources (Griffin and Guez, 2015, Visalberghi and Addessi, 2000; but see; Stöwe, Bugnyar, Heinrich, & Kotrschal, 2006). Alternatively, studies of captive house sparrows, *Passer domesticus* (Liker & Bókony, 2009) and wild flocks of great tits, *Parus major* (Morand-Ferron & Quinn, 2011) have argued in favour of the ‘skill pool’ or ‘pool of competence’ hypothesis, whereby group size effects on innovation are driven by greater phenotypic diversity within larger groups (Giraldeau, 1984, Giraldeau and Lefebvre, 1986). Larger groups are more likely to have a greater variety of individuals in terms of age, dominance rank, motor skills and neophobia, all of which have been found to influence innovative behaviour (Biondi et al., 2010, Griffin and Guez, 2014, Keynan et al., 2015, Thornton and Samson, 2012). However, the evidence for ‘pool of competence’ effects in wild animal groups is limited to a single study (Morand-Ferron & Quinn, 2011).

In contrast to studies reporting positive associations between group size and innovation, Griffin et al. (2013) and Overington et al. (2009) found that larger group sizes inhibit innovative behaviour. Captive Carib grackles, *Quiscalus lugubris*, were slower to produce innovative solutions to a novel foraging task when in the presence of conspecifics (Overington et al., 2009). Similarly, the innovative propensity of wild-caught Indian mynas, *Acridotheres tristis*, was greater when alone than in the presence of five conspecifics, or in pairs (Griffin et al., 2013). In addition, group size failed to explain the likelihood of wild meerkats, *Suricata suricatta,* interacting with (or solving) foraging tasks (Thornton and Malapert, 2009a, Thornton and Samson, 2012). Likewise, solitary ravens, *Corvus corax*, were more likely to approach novel objects than ravens in dyads or groups (Stöwe et al., 2006). Thus, although large groups may provide individuals with some benefits, increased levels of competition, scrounging and aggression could also reduce opportunities for innovation as group size increases (Overington et al., 2009).

Although the effect of group size on the emergence of innovative behaviour is poorly understood, the effect of group size on the subsequent spread of novel information has received even less attention, particularly in wild animal populations. Once an innovative behaviour has emerged, naïve group members may learn it from experienced conspecifics (Aplin et al., 2015). Evidence for social learning is well documented across a wide range of taxa in both captive and wild conditions (for a comprehensive review see Hoppitt & Laland, 2013). However, the potential effect of group size on the rate at which novel information spreads through a group is unclear. Evidence from studies on humans suggests that innovations are transmitted more rapidly and effectively in larger groups (Derex et al., 2013, Mithen, 1994); consequently, we predict that novel information will spread more rapidly in larger groups of nonhuman animals via social learning.

In this study, we examined the relationship between group size and the emergence and subsequent spread of innovation in a wild population of colour-ringed and habituated Australian magpies (Western Australian subspecies *Cracticus tibicen dorsalis*; hereafter referred to as ‘magpies’). Magpies are large (250–400 g) cooperatively breeding passerines that live in territorial groups ranging from two to 12 adults, in which multiple individuals of both sexes contribute to rearing offspring and territorial defence (Ashton et al., 2018, Edwards et al., 2015, Hughes et al., 2003, Kaplan, 2008, Mirville et al., 2016, Pike et al., 2019). Unlike other subspecies of Australian magpie, sex can be determined visually in Western Australian magpies because they are sexually dichromatic (Ashton et al., 2018). We presented a novel foraging task to magpie groups of differing sizes, whereby a food reward could be accessed by pushing a self-shutting sliding door either left or right. We predicted that in larger groups (1) innovations would arise more rapidly and (2) innovative behaviour would spread more rapidly, facilitated by social learning.

## Methods

### Study Site and Population

The study took place in the urban grassland areas of Guildford, Western Australia, during the breeding season from July to December 2014. The study population consists of 16 groups, ranging in size from two to 11 individuals (excluding fledglings). The study population is habituated to human presence, allowing detailed behavioural observations (from <2 m) and the presentation of novel foraging tasks (Ashton et al., 2018). In 11 of the 16 groups (hereafter referred to as ‘core groups’, *N* = 65 birds), the majority of individuals are ringed, allowing individual identification. The remaining five groups are either partially ringed or contain no ringed individuals (*N* = 37 birds). Over the course of the study period all group sizes remained the same. To determine the size and composition of unringed groups we waited until they were foraging in open parklands. Multiple trips confirmed stable group size in both ringed and unringed groups. All 16 groups were included in analyses investigating the emergence of innovative behaviour, as individual identification was not necessary. However, groups containing mainly unringed individuals were excluded from further analyses investigating the spread and social transmission of innovative behaviour because individual identity could not be reliably confirmed in experimental trials.

### Novel Foraging Task

**Figure 1:**
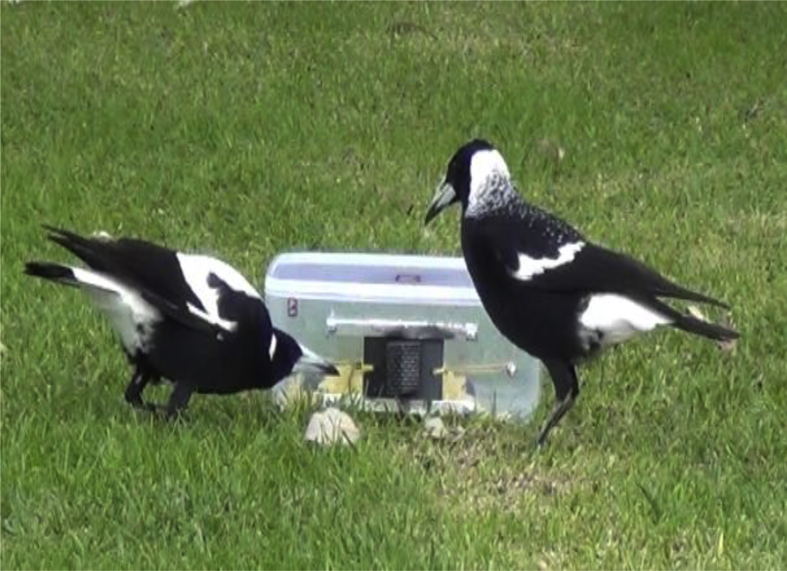
Novel foraging task. Food rewards could be extracted by pushing the self-shutting sliding door either left or right.

To investigate the emergence of innovative behaviour, we presented 16 groups of magpies with a novel foraging task, similar to that used on other bird species previously (Aplin et al., 2015). To determine the role of group size in the natural emergence and spread of innovative behaviour we did not train specific individuals to act as ‘demonstrators’, unlike previous experiments (Aplin et al., 2015, Gunhold et al., 2014, Gunhold et al., 2014, Thornton and Malapert, 2009b); for example Aplin et al. (2015) trained individuals on a specific solving technique (either pushing left or right). The task consisted of a transparent plastic box containing grated mozzarella cheese as a food reward (Fig. 1). The reward could be obtained by pushing a self-shutting sliding door either left or right. Elastic bands caused the door to reset to the central (closed) position immediately after being released, thus preventing others from scrounging the food reward accessed by a ‘solver’ individual. To avoid devices being monopolized by a single dominant individual, we presented two identical devices to each of the 16 groups, in open parkland areas where they routinely forage. Devices were only presented when all members of the group were present within 20 m and were placed 2 m apart in the middle of an area where the group was foraging. Each device contained sufficient food such that it did not become depleted during trials. All experimental trials were carried out as close to sunrise as possible (between 0430 and 0700 h according to season). Experimental trials were recorded using a Sony Handycam (model HDR-XR260VE) and transcribed via video analysis using the Cybertracker program (https://www.cybertracker.org) on an Asus Google Nexus 7 tablet. Of the 11 core groups, each ringed individual's behaviour was transcribed separately for each experimental trial at each group. All activity around the device was recorded, including time spent oriented towards the device, whether the bird made contact with the device, whether it attempted to obtain the food reward (both successful and unsuccessful attempts, i.e. pecking at the transparent box), and, if successful, in what direction it pushed the door. Any aggressive and submissive interactions between individuals during the trial were also recorded. Aggressive actions were defined as any dominant behaviour directed from one individual to another (e.g. pecking and chasing behaviour). Submissive behaviours included birds vocally and physically submitting (rolling onto their backs), as well as retreating from an approaching individual. Since neophobia may play an important role in the emergence of innovative behaviour, for each trial we also recorded each individual's latency to make contact with the device after coming within 5 m of it. In addition, the identity of birds observing other individuals interacting with the device (whether they solved it or not, in what direction they pushed the door) was also recorded. Individuals were quantified as observing if their head was oriented towards an individual interacting with the task, they were within 10 m of the individual, their body was oriented towards the individual, they had an uninterrupted line of sight and they were not engaging in any other activities at that time (Samson & Manser, 2016). To determine the interrater reliability of quantifying ‘observing’, 10% of trials were recorded by two people (*N* = 22 novel foraging task trials). Intraclass correlation coefficients (ICC) indicated a high level of reliability (ICC = 0.982, *P* < 0.001, *N* = 22 trials). Experimental trials were terminated after 15 min, or when any group member left the trial (i.e. when an individual that had been interacting with the task moved more than 20 m away from either of the two devices). Each group was presented with the devices until every individual in the group had learned to access food (range 1–7 trials, mean ± SE = 3.1 ± 0.49), with 24 h between each presentation. The order in which groups were initially presented with the device was selected randomly. Once initially selected, trials were carried out on consecutive days at each group. To determine whether reduction in the need for antipredator vigilance may be the cause of a possible relationship between group size and the emergence of innovative behaviour, we monitored antipredator behaviour (mobbing of predators, alarm calling, sentinel duty), but no such behaviour was observed during any experimental trial. In addition, behavioural focal follows were collected at the study site during the experimental period (20 min behavioural activity focal follows, carried out on all individuals multiple times per week; for further details of focal follows see Edwards et al., 2015), and the frequency of antipredator behaviour recorded was very low (mean ± SE = 0.067 ± 0.018 antipredator events per 20 min focal follow). Australian magpies are a large passerine with few natural predators (Johnstone & Storr, 2004); it is therefore unsurprising there were no antipredator behaviours observed during experimental trials.

### Ethical Note

All methods were performed in accordance with the University of Western Australia's guidelines and regulations and were approved by the University of Western Australia Animal Ethics Office (ref: RA/3/100/1272).

### Statistical Analyses

#### Effect of group size on innovation emergence and spread

First, to determine whether innovative behaviour emerges more rapidly in larger groups we fitted a nonlinear, exponential regression between group size and the time taken for innovative behaviour to emerge (i.e. time (s) until the task was first solved within each group).

Second, we used a Cox proportional hazards regression model to investigate the effect of group size on the time taken for innovative behaviour to spread after initial emergence. A Cox proportional hazards regression was used so we could account for the fact that some individuals could have learnt the innovative behaviour if given more time. The response term used was the time (s) taken for each individual to learn the innovative behaviour after initial emergence within the group. Following Harrison et al. (2018) we adopted a hypothesis-driven modelling approach, rather than constructing a global model, whereby we carried out a series of models each investigating the effect of a different ecological variable. Explanatory terms included were group size, the number of innovators in the group at the time of solving (multiple innovators are possible in a group if individuals solved the task without having observed any other individual attempt the task), the sex ratio of adults in the group, and the number of aggressive and submissive interactions between individuals during experimental trials. It is also possible solving time might be influenced by differences in neophobia, so latency to interact with the task was also included as an explanatory term. We clustered the observations around group identity to account for interdependence in the data, because solving times within groups might be correlated. The initial innovators were removed from Cox proportional hazards regression models to ensure that we were only examining the spread rather than the initial emergence of innovation. We did not include age in analyses as we do not know the exact fledging date of the majority of adults in the study population, and too few juveniles and fledglings were tested (*N* = 7) to include age as a categorical term (adult versus juvenile).

#### Social transmission of door opening preferences

To examine whether magpies showed any consistent side biases between pushing left or right on the device, we ran a binomial test on the initial innovators in each group. Only initial innovators were used in this analysis to control for social information use, which may influence the direction pushed for subsequent solvers.

To determine whether social information use influenced the direction in which observer birds pushed the door, we ran a generalized linear mixed model to determine whether observers were more likely to push the door in the same direction than the individual they first observed. The response term used was the direction first pushed by the solver (binary response term, right=1, left=0); explanatory terms were the direction pushed by the first individual they observed, sex (of the observer) and group size. Group identity was included as a random term. Analyses were conducted using IBM SPSS Statistics software (version 22, IBM, Armonk, NY, U.S.A.) and the survival package (Therneau, 2015) in R (v.3.1.1, http://www.r-project.org) was used for the Cox proportional hazards regression.

## Results

### Do Innovations Emerge More Rapidly in Larger Groups?

**Figure 2:**
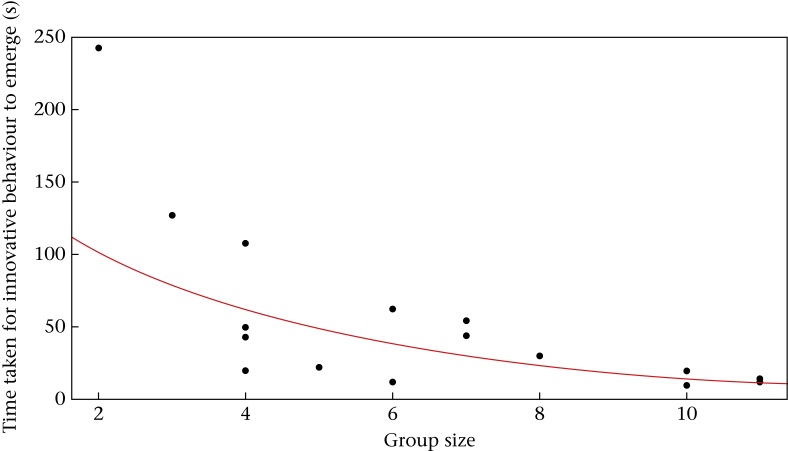
The time (s) taken for innovative behaviour to emerge (once individuals first interact with the task) in relation to group size.

We recorded a total of 1050 attempts to gain access to the food reward, including pecking and pushing the door, by 65 individuals across the 11 core groups. Of these 65 birds, 50 were successful in accessing food (rate of success = 76.92%). Of these 50, 21 were never seen to observe other birds solving, and so were classed as innovators. The number of innovators per group ranged from one to four (mean ± SE = 2.18 ± 0.33) and group size correlated positively with the number of innovators per group (Spearman correlation: *r*_S_ = 0.637, *N* = 11, *P* = 0.035). At the group level, there was a nonlinear decline in the time taken for the innovative behaviour to emerge, with innovative behaviour emerging more quickly in larger groups (exponential regression: *r* = 0.559, *P* = 0.001, Akaike information criterion, AIC = 146.7; Fig. 2). An exponential model fitted the data better than a linear model (linear regression: *r* = 0.428, *N* = 16, *P* = 0.006, AIC = 172.91). This pattern remained when the data point for the group size of two was removed (exponential regression: *r* = 0.475, *P* = 0.01, AIC = 132.85; linear regression: *r* = 0.395, *P* = 0.01, AIC = 146.98).

### Do Innovations Spread More Rapidly in Larger Groups?

**Figure 3:**
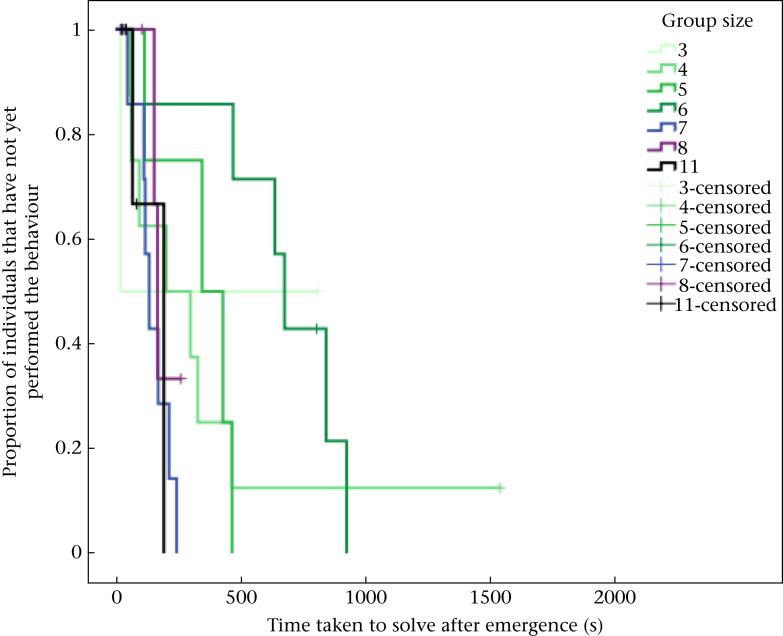
Survival curves showing the effect of group size on the spread of innovative behaviour within groups.

A Cox proportional hazards model revealed there was a significant effect of group size on the spread of innovative behaviour after the initial innovation in each group: innovative behaviour spread more quickly in larger groups (Table 1, Fig. 3). The number of innovators at the time of solving did not have a significant influence on the spread of innovative behaviour. Latency to approach the task and the number of aggressive or submissive interactions also did not influence the spread of innovative behaviour (Table 1).

**Table 1:** Survival models (Cox's proportional hazards regression) for the proportion of group members that learnt the innovative behaviour

Variable	±SE	*Z*	*P*
Group size	**0.09**	**4.41**	**<0.001**
No. of innovators at time of solving	0.18	-1.41	0.158
No. of aggressive and submissive interactions	0.08	-1.09	0.273
Latency to interact	0.003	0.43	0.666
Sex ratio	1.328	1.19	0.233

Statistically significant term is in bold. *N* = 54 individuals from 11 groups of seven different group sizes.

### Does Social Information Use Play a Role in the Spread of Innovations?

Of the 11 core groups over all the trials, 44.62% of birds (*N* = 29) solved the task after observing another individual successfully solve it, 32.31% of birds (*N* = 21) solved the task having not observed any individuals interact with the task and 13.85% of birds (*N* = 9) failed to solve the task. We do not know whether the unringed birds (9.23%, *N* = 6) solved the task. When the innovative behaviour first emerged in groups, innovators were no more likely to push left (*N* = 3 individual innovators) or right (*N* = 8), suggesting there was no intrinsic, population level side bias (binomial test: *N* = 11, *P* = 0.227). However, individuals that solved the task after observing a successful attempt were significantly more likely to push the door in the same direction as the solver they first observed, rather than the alternative direction (Table 2). This trend remained when the initial observation was the door being pushed left (*N* = 7 of 8 individuals pushing the same way [87.5%], binomial test, *P* = 0.07) and right (*N* = 17 out of 21 individuals pushing the same way [80.95%]; binomial test: *P* = 0.007). The trend for pushing the same way when the initial observation was left was not significant; however, the proportion of individuals pushing the same way was high (higher than when the initial observation was right), suggesting the lack of significance may be due to a small sample size.

**Table 2:** GLMM model investigating factors affecting the direction first pushed by observers at the device, including the full model set (top) and top set (bottom)

	QICc		ΔQICc	
Full model				
Direction first observed	30.479		0	
Basic	40.496		10.017	
Group size	42.333		11.854	
Sex	42.593		12.114	
Top set	Estimate	SE	Confidence interval	*P*
Direction first observed	3.393	1.067	1.302, 5.483	0.001

QICc = corrected quasi-likelihood under the independence model criterion. The top set includes models within 2 QICc values of the best model. Group identity was included as a random term. *N* = 29 individuals.

## Discussion

Positive relationships between group size and the emergence of innovative behaviour have been suggested as a possible benefit of living in larger groups (Giraldeau, 1984). In accordance with this prediction, we found an asymptotic effect of group size on the time taken to solve the task, with individuals in larger groups solving a novel foraging task faster than those in smaller groups. The differences in the time taken to learn the innovative behaviour (and the time taken for the innovative behaviour to spread) between small and large groups are relatively small compared to other studies investigating the spread of innovative behaviour (e.g. Liker and Bókony, 2009, Aplin et al., 2015). However, note that although the time periods are small, this is an experimental study using a relatively simple task. Where problems are more challenging and require greater skill or experience to master, the effect of group size on the emergence and spread of innovative behaviour might be larger. It is also possible the asymptotic relationship between group size and the emergence of innovative behaviour was a product of the simplicity of the task. Had the task been harder, performance might not have been bound by quick solving times as it was in our study, which would allow larger group sizes to innovate more quickly than medium-sized groups. Such a scenario could create a linear relationship between group size and the emergence of innovative behaviour. Future studies may avoid this issue by presenting multiple tasks of varying difficulty, or a single more difficult task. To our knowledge, only two other studies have reported positive effects of group size on the emergence of innovative behaviour in the context of novel foraging tasks (Liker and Bókony, 2009, Morand-Ferron and Quinn, 2011; although see the literature on collective decision making, e.g. ; Sumpter, Krause, James, Couzin, & Ward, 2008). Our findings thus add to the existing evidence that group size effects may play a critical role in driving behavioural innovations in wild populations.

In previous research, positive relationships between group size and the emergence of innovative behaviour have been postulated to result from the ‘skill pool’ effect, where larger groups have more individuals with a greater range of traits at their disposal, enabling them to solve novel problems more rapidly (Giraldeau, 1984, Morand-Ferron and Quinn, 2011). However, it remains possible that an exponential relationship between group size and time taken to innovate could be generated by statistical probability, without the need to invoke a skill pool explanation. If, for instance all individuals have an equal, set probability of solving a task at a given time step, then the decrease in time taken for innovative behaviour to emerge scales exponentially with group size. This is because the larger the group is, the greater the probability that there will be an individual who will solve it more quickly than the set probability at the given time step (Hoppitt, Kandler, Kendal, & Laland, 2010 made similar arguments concerning the use of sigmoidal curves as diagnostics of social learning). Therefore, while our current results appear consistent with the predictions of the pool of competence hypothesis, it is not possible to conclude whether this relationship emerged due to a skill pool effect or simple statistical probability. To address whether increased phenotypic diversity per se facilitates the speed or likelihood of innovation, the best approach may be to compare the emergence of innovative behaviour between groups of the same size with different compositions of individuals in terms of age, sex, dominance status or personality. Nevertheless, regardless of the precise mechanism underpinning the effect, our findings still indicate that the rapid emergence of solutions to novel problems may be a substantial benefit of living in large groups.

Explanations other than the skill pool hypothesis have been suggested for the observed relationships between group size and the emergence of innovative behaviour. For instance, positive relationships could be the result of an antipredator vigilance effect in larger groups, allowing more time for exploration and innovative behaviour (Griffin & Guez, 2015). During our experimental trials we recorded no antipredator behaviour (e.g. mobbing of predators, alarm calling, sentinel duty), suggesting that antipredator effects are unlikely to account for the relationship between group size and the emergence of innovative behaviour observed in Australian magpies. Likewise, Liker and Bókony (2009) found no evidence of antipredator vigilance on the success rate at innovative problem-solving tasks. It is also possible that indirect effects of group size on neophobia may drive the group size–innovation relationship. However, latency to contact the task did not predict performance. Alternatively, the recent finding that Australian magpies from larger groups have greater general cognitive performance compared to magpies from smaller groups (Ashton et al., 2018) suggests that cognition may play an important role in the relationship between group size and the emergence of innovative behaviour. If larger groups are composed of individuals with greater cognition, and performance on the task is underpinned by cognitive traits, this may explain faster solving times in larger groups.

Conversely, there is also evidence to suggest that larger group sizes can inhibit innovative behaviours (Griffin et al., 2013, Overington et al., 2009). It has been speculated this may be due to increased antagonistic behaviour in larger groups (Overington et al., 2009). However, in our study the frequency of aggressive and submissive interactions between individuals at the experimental device had no effect on the time taken for individuals to learn the innovative behaviour. Additionally, both Overington et al. (2009) and Griffin et al. (2013) found aggressive behaviour did not underpin the reported negative relationships between group size and innovative behaviour. In combination, this suggests that levels of aggression do not play an important role in the relationship between group size and the emergence of innovative behaviour, whether this relationship is positive or negative.

The rapid emergence of innovative behaviour in large groups will be particularly beneficial if other group members can learn from the initial innovator (note that other group members may innovate themselves). We found that innovative behaviour spread more rapidly in larger groups, and evidence of side copying on the task indicates that this was probably facilitated through social learning. This suggests larger group sizes promote not only the initial emergence of innovations, but also the subsequent transmission among group members. Theoretical and empirical research suggests that larger population sizes played a critical role in the accumulation of cultural knowledge in human societies (Muthukrishna, Shulman, Vasilescu, & Henrich, 2014). Our results suggest that this may also be the case in group-living nonhuman animals. The positive relationship between group size and the number of innovators within a group may lead one to hypothesize multiple innovation events in larger groups may facilitate the spread of innovative behaviour. However, there was no effect of the number of innovators at the time of solving on the spread of innovative behaviour. Information may spread particularly rapidly in larger groups due to the greater frequency of social interactions (Mithen, 1994), but of course transmission rates will also be influenced by the particular structure of social networks (Franz & Nunn, 2009). Investigating the interplay between social group size and network structure in social transmission dynamics in natural animal populations is therefore an important priority for future research.

In conclusion, our study provides evidence for a positive relationship between group size and innovation in wild animals. Furthermore, our findings show that, as suggested by studies of human cultural transmission (Derex et al., 2013, Kempe and Mesoudi, 2014, Mithen, 1994, Muthukrishna et al., 2014), novel information spreads more rapidly in larger groups. Together, our results provide a rare link between the emergence and spread of novel information in a social animal in its natural environment.

## Author Contributions

B.J.A. carried out the fieldwork and statistical analyses, drafted the manuscript and participated in the design of the study; A.T. and A.R.R. participated in the design of the study and helped draft the manuscript. All authors gave final approval.

## Conflicts of Interest

We have no competing financial interests.
